# Tibial transverse transport promotes wound healing in diabetic foot ulcers by stimulating endothelial progenitor cell mobilization and homing mediated neovascularization

**DOI:** 10.1080/07853890.2024.2404186

**Published:** 2024-09-16

**Authors:** Weiqing Tian, Lan Zhang, Yongjun Wang, Ligong Lin, Wei Jiang, Guangming Dai, Bo Feng

**Affiliations:** Department of Orthopedics, Baogang Hospital of Inner Mongolia Autonomous Region, Baotou, P. R. China

**Keywords:** Diabetic foot ulcers, Tibial transverse transport, endothelial progenitor cells, angiogenesis, wound healing

## Abstract

**Background:**

Diabetic foot ulcers (DFUs) are a common and serious complication of diabetes, often leading to amputation and decreased quality of life. Current treatment methods have limited success rates, highlighting the need for new approaches. This study investigates the potential of tibial transverse transport (TTT) to promote wound healing in DFUs.

**Methods:**

To test this hypothesis, the study used New Zealand White rabbits to establish a diabetic model and simulate foot ulcers, followed by the treatment of unilateral TTT or bilateral TTT. The study employed histological analysis, flow cytometry, ELISA, and qPCR to assess the impact of TTT on tissue repair and endothelial progenitor cell (EPC) mobilization and homing, aiming to understand the underlying biological processes in wound healing.

**Results:**

TTT significantly enhanced wound healing in diabetic rabbit foot ulcers. Specifically, bilateral TTT led to complete wound healing by day 19, faster than the unilateral TTT group, which healed by day 26, and the sham operation group, which nearly healed by day 37. Histological analysis showed improved tissue architecture, collagen deposition, and neovascularization in TTT-treated groups. Furthermore, TTT treatment resulted in a significant increase in VEGFR2 expression and VEGFR2/Tie-2 positive cells, particularly in the bilateral group. These findings were corroborated by qPCR results, which showed increased expression of VEGFA and CXCL12 by TTT. **Conclusions:** TTT may be a promising treatment for DFUs, significantly enhancing wound healing by stimulating EPC mobilization and homing mediated angiogenesis. This novel approach could substantially improve treatment outcomes for diabetic patients with chronic foot ulcers.

## HIGHLIGHTS

1. TTT accelerates wound healing in diabetic rabbit instep ulcers, with both unilateral and bilateral surgeries effective, and bilateral TTT showing enhanced efficacy.
2. TTT boosts angiogenesis and collagen fiber formation, leading to increased granulation tissue and re-epithelialization of wounds.
3. TTT induces the mobilization and homing of endothelial progenitor cells to promote angiogenesis and wound healing.

## Introduction

Diabetic foot ulcers (DFUs) are commonly caused by a combination of peripheral neuropathy, leading to increased local stress and peripheral vascular disease [1]. DFUs represent one of the most severe and costly complications of diabetes, affecting approximately 15% of diabetic patients at some point in their lives [2]. These chronic wounds result from a combination of factors, including neuropathy, vascular disease, and immune dysfunction [3]. Neuropathy reduces the patient’s perception of pain and trauma, while vascular disease diminishes blood supply to the feet, making the feet prone to injury and slow to heal [4]. Current DFU treatments include debridement, infection control, glycemic optimization, and diverse dressings [5]. However, these treatments face challenges due to the impaired healing ability of diabetic patients, leading to complex and lengthy healing processes even for minor wounds [6,7]. The high prevalence, disability, mortality rates, and lack of effective treatment make the management of diabetic foot a challenging clinical problem [8,9].

Tibial transverse transport (TTT), an emerging DFU treatment, has shown promise in improving limb salvage rates, wound healing, sensation, and blood circulation in diabetic foot [10,11]. The mechanisms of TTT’s effectiveness remain unclear. Angiogenesis is a key process in wound healing, especially in DFUs where blood supply is insufficient, and the formation of new vessels is crucial for restoring tissue perfusion and promoting wound healing [12]. Moreover, in the healing process of diabetic wounds, endothelial progenitor cells (EPCs) are mobilized into circulation and recruited to the wound tissue by responding to hypoxia and inflammation, and aiding neovascularization and wound healing by secreting various growth factors and cytokines [3]. In addition, reduced VEGFA and subsequent poor vascular growth are key factors in DFU non-healing [13]. Studies indicate that VEGFA can promote C-X-C motif chemokine ligand 12 (CXCL12), regulating the mobilization and homing of EPCs [14]. Evidence suggests that fibroblasts may be a key source of VEGFA and CXCL12 production in skin wounds, indicating that fibroblast secretion of VEGF and CXCL12 promotes EPC mobilization, mediating angiogenesis and wound healing [15]. In our previous study, TTT can accelerate wound healing of chronic foot ulcers through the VEGFA/CXCL12 pathway [16].

In summary, it is hypothesized that TTT may promote the expression of VEGFA and CXCL12, thereby affecting the mobilization and homing of EPCs, mediating neovascularization, and healing of DFU wounds.

## Materials and methods

### Animal models and treatment

In this study, six-month-old healthy New Zealand White rabbits (male; weighing 2.5-3.0 kg) were used, purchased from the Experimental Animal Center of Southern Medical University (Guangzhou, China). All animal experiments were conducted in accordance with the guidelines of the International Animal Protection Association and approved by the Animal Ethics Committee of Inner Mongolia Baogang Hospital (Baotou, China). Before the commencement of the experiments, all rabbits were acclimatized under standard conditions for one week. The groups were divided into: sham operation (*n* = 5), unilateral TTT (*n* = 5), and bilateral TTT (*n* = 5). After grouping, a diabetic model was established by injecting streptozotocin (STZ, 65 mg/kg, dissolved in freshly prepared citrate buffer) daily for three consecutive days. The criterion for successful establishment of the diabetic models was a fasting blood glucose level consistently above 16.7 mmol/L. After establishing the diabetic models, the instep surfaces of the rabbits’ hind feet were selected as the site for the ulcer models. Under local anesthesia, standardized full-thickness skin defects of 1.5 cm diameter were created using a circular skin punch in the designated area to simulate ulcers. Immediately after creating the ulcers, the wounds were cleaned with sterile saline and covered with sterile dressings.

For treatment, unilateral TTT or bilateral TTT was performed in DFU rabbits. The surgical procedure involved making an incision over the tibia, performing a controlled transverse osteotomy, and stabilizing the bone segments with an external fixator to facilitate gradual distraction. The surgical protocol for animal surgery was modified from Yang et al. [17] and Chen et al. [18].

And we used general anesthesia with ketamine (35 mg/kg) and xylazine (5 mg/kg), as recommended by the study [19]. Post-operative pain was managed with meloxicam (0.2 mg/kg) administered subcutaneously once daily for three days by following protocols [20]. Rabbits were closely monitored, and additional analgesics were provided as needed. To prevent infections, enrofloxacin (10 mg/kg) was given subcutaneously immediately post-surgery and continued once daily for five days [21].

During the experiment, the rabbits’ weight, blood glucose levels, and ulcer area were monitored daily using digital photography and image analysis software. Ulcer tissue samples were taken weekly for histological analysis to assess the inflammatory response and healing process. Photos of the wounds were taken one day before surgery and on days 3, 7, and 14 post-surgery to calculate the wound healing rate and duration, and the skin tissue or blood samples were collected for further detections.

### Hematoxylin and eosin (HE) staining

Initially, fixed and embedded skin tissue samples (days 7 and 14 post-surgery) were sectioned at 4 μm thickness and affixed to slides, which were then placed in a 60 °C drying oven for fixation. The sections underwent a series of treatments beginning with xylene for deparaffinization, followed by a graded ethanol series for dehydration—commencing with absolute ethanol, then 95%, and finally 70% ethanol. Subsequent to dehydration, the sections were stained with Harris’s hematoxylin to facilitate the visualization of nuclear and cytoplasmic components. After staining, the sections were thoroughly rinsed and subjected to a final dehydration step. Upon completion of the dehydration process, a mounting medium was applied, and the sections were cover-slipped and left to dry. These prepared slides were examined under an optical microscope, and images were taken to scrutinize the pathological changes in the wound skin.

### *Masson*’*s trichrome staining*

The skin tissue samples (days 7 and 14 post-surgery) used in the experiment were obtained from a previously described diabetic rabbit foot ulcer models. At various time points during the experiment, the rabbits were euthanized, and skin samples from around the ulcers were collected for analysis. The samples were fixed in 4% paraformaldehyde solution for 24 h, then dehydrated and embedded in paraffin. Continuous sections of 4 μm thickness were cut using a rotary microtome and mounted on slides for further processing. The paraffin sections were deparaffinized in xylene, rehydrated through graded alcohols, and washed with water [22]. The sections were then stained with Weigert’s iron hematoxylin solution for 5 min for nuclear staining, treated with phosphoric acid solution for 1 min, and washed again with water [23]. Subsequently, the sections were stained in Masson’s Trichrome solution for 5-10 min, washed with water, and treated with 1% acetic acid solution for 1 min. Finally, the sections were dehydrated in graded alcohols, cleared in xylene, and mounted. The collagen fiber content and distribution in the ulcer margin tissue were analyzed under a microscope to assess collagen deposition during the healing process of DFUs. Image analysis software was used to quantitatively analyze the images and record the area ratio of collagen fibers.

### Immunofluorescence

For CD31, VEGFA, and VEGFR2 detection, the prepared skin tissue sections (days 7 and 14 post-surgery) were incubated with the primary antibodies anti-CD31 (GTX20218, 1:100, GeneTex, Irvine, CA, USA), anti-VEGFA (bs-1313R, 1:200, Bioss, Beijing, China), and anti-VEGFR2 (bs-10412R, 1:100, Bioss) at 4 °C overnight, respectively. After the incubation with secondary antibody, the sections were imaged using a fluorescence microscope (EVOS FL, Life technologies, USA) and the levels of CD31, VEGFA and VEGFR2 were calculated using ImageJ software.

### Flow cytometry analysis

On the days 7 and 14 post-surgery, blood samples were collected in tubes coated with 0.1 M EDTA-2Na. Anti-VEGFR2 (bs-10412R, Bioss) and anti-Tie-2 (AF313, R&D Systems, Minneapolis, MN, USA) antibodies were used to stain the peripheral blood at 37 °C for one hour. False-positive cells were exclude using the isotype control IgG. Next, the peripheral Blood was lysed and fixed in FACS^TM^ lysing solution (BD Biosciences, San Jose, CA, USA) for 5 min, followed by the washing with PBS and centrifugation (at 300 *g*) for 5 min. Then, the peripheral Blood were suspended in 400 µL PBS for flow cytometry analysis. VEGFR2 and Tie-2 double-positive were analyzed using the FACS Canto-II flow cytometer (BD Biosciences) to assess EPCs.

### Enzyme-linked immunosorbent assay (ELISA)

Quantitative determination of serum cytokines (on the one day before surgery and days 3, 7, and 14 post-surgery) was conducted using commercially available enzyme-linked immunosorbent assay (ELISA) kits. Specifically, rabbit VEGFA ELISA kit (kl-deim-00300, Ke Lei Biological Technology Co., Ltd., Shanghai, China) and CXCL12 ELISA kit (CSB-E12656Rb, Cusabio, Wuhan, China) were assayed. All procedures were meticulously performed in strict accordance with the manufacturer’s protocols provided with the kits.

### Quantitative real-time polymerase chain reaction (qPCR)

The total RNA was extracted from the wound skin tissues (on the one day before surgery and days 3, 7, and 14 post-surgery) using TRIzol reagent (Thermo Fisher Scientific, Waltham, MA, USA) and cDNA was synthesized using a Transcriptor First Strand cDNA Synthesis Kit (Roche, Basel, Switzerland). Quantitative real-time PCR was performed using LightCycler 480 SYBR Green I Master Mix (Roche). The 2^−ΔΔCt^ method to assess the relative expression level of genes and normalized it to β-actin. The primer sequences were listed as follows: VEGFA, 5′-CCT TCA CCT ACT GTC ATC TGC TTC C-3′ (forward), 5′-GCT ACT ACT TCG TCC ACT CTT CTT CC-3′ (reverse); CXCL12, 5′-CCC ACC ATC TAC TCC ATC A-3′ (forward), 5′-GAA ATC GGG AAT AGT CAG C-3′ (reverse); β-actin, 5′-TCC TGC GTC TGG ACC TGG-3′ (forward), 5′-GCC CGA CTC GTC ATA CTC C-3′ (reverse).

### Statistical analysis

Data are presented as mean ± standard deviation (SD). Statistical analysis was performed using GraphPad Prism 8.0 software (GraphPad Software, Inc.). Comparisons between multiple groups were made using one-way analysis of variance (ANOVA) with Tukey’s post hoc test. Statistical significance was determined as indicated in the figure legends, with a P-value of less than 0.05 considered statistically significant.

## Results

### TTT enhances wound healing in diabetic rabbit foot ulcers

**Figure 1.**
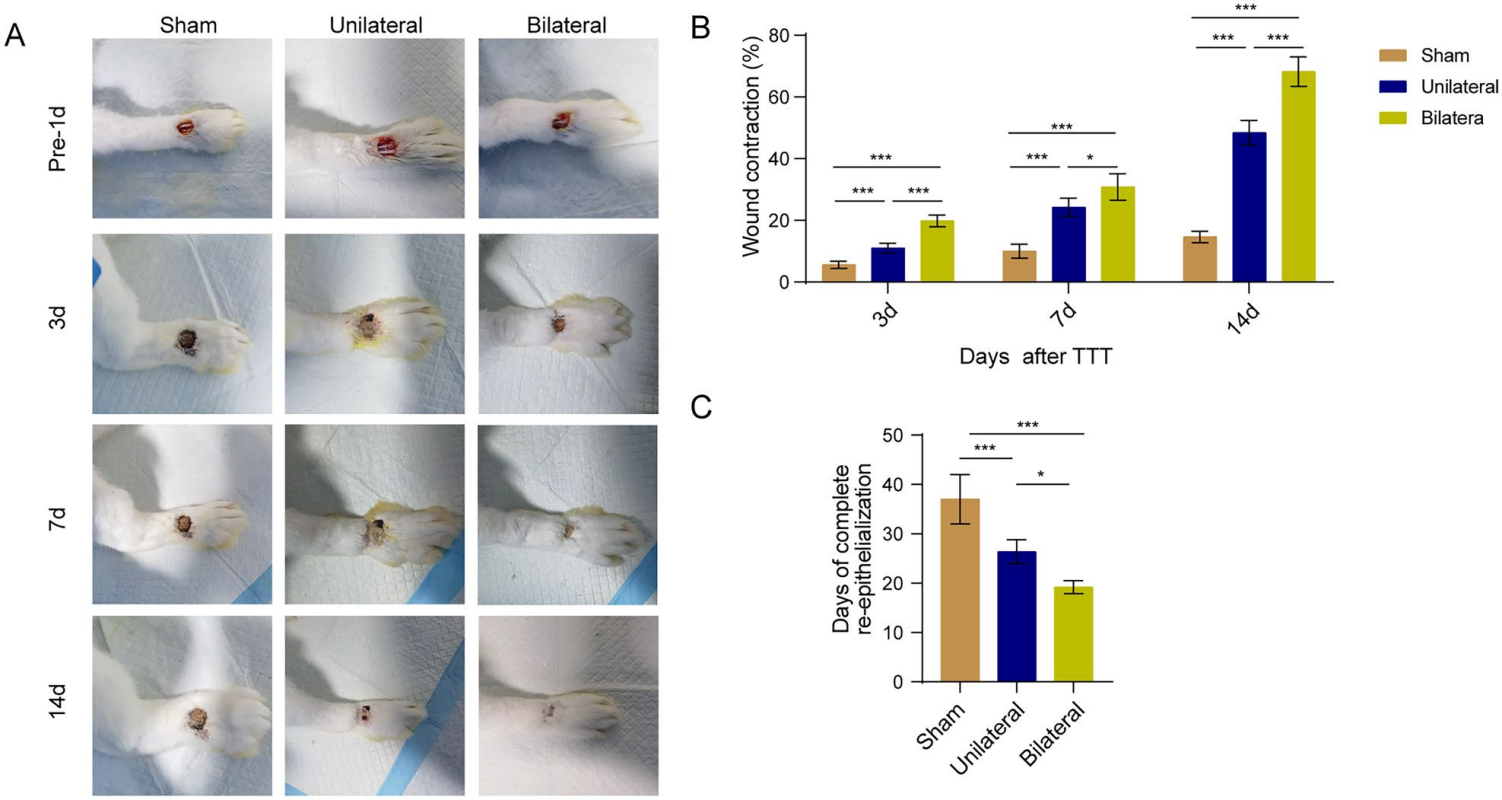
TTT enhances wound healing in diabetic rabbit foot ulcers. (A) Photographs of foot wounds. (B) Statistical analysis of wound healing rate. (C) Statistical analysis of ulcer wound healing days. *, *p* < 0.05, ***, *p* < 0.001 compared to the sham group or unilateral TTT group.

Initially, we investigated the effect of TTT on ulcer wounds in diabetic rabbit foot ulcer models. The results revealed that both unilateral and bilateral TTT surgeries significantly promoted wound healing in diabetic rabbits, with bilateral TTT showing superior efficacy (Figure 1A, B). Moreover, by day 37, wounds in the sham operation group were nearly completely healed, while those in the unilateral TTT group healed completely around day 26, and the bilateral TTT group around day 19 (Figure 1C). These results suggest that TTT surgery can significantly shorten the wound healing time in diabetic rabbit foot ulcers. In summary, TTT shows a beneficial therapeutic effect in healing diabetic rabbit foot ulcer wounds.

### The effect of TTT on angiogenesis and collagen fiber formation

**Figure 2.**
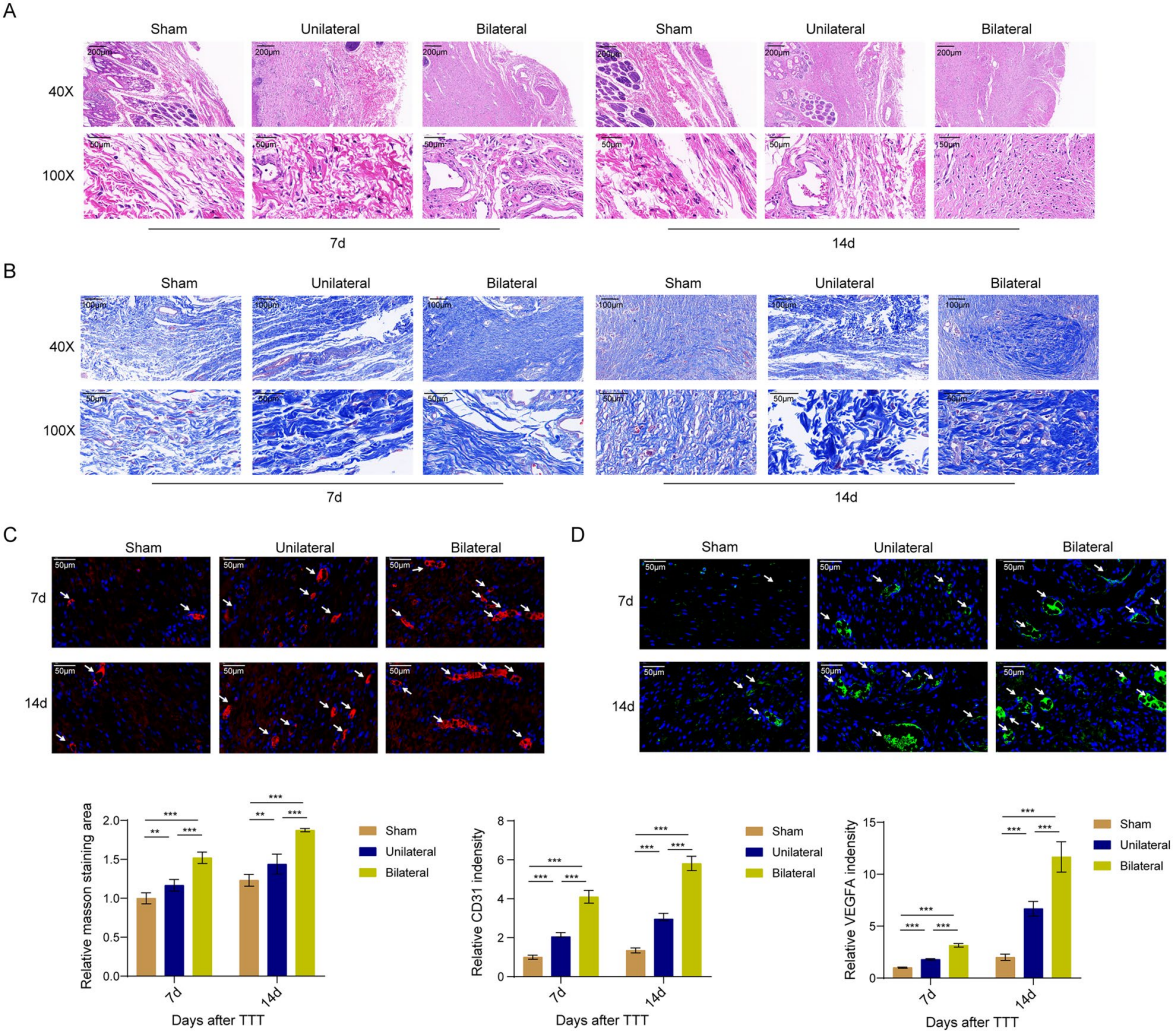
The effect of TTT on angiogenesis and collagen fiber formation. (A) HE staining for granulation tissue deposition and epidermal regeneration. Scar bar = 200 or 50 μm. (B) Masson’s trichrome staining for collagen fiber formation. Scar bar = 100 or 50 μm. (C) Immunofluorescence for CD31 expression changes, anti-CD31 antibody and DAPI to the nuclei, and the arrow to indicate positive staining. Scar bar = 50 μm. (D) Immunofluorescence for VEGFA expression changes, anti-VEGFA antibody and DAPI to the nuclei, and the arrow to indicate positive staining. Scar bar = 50 μm. **, *p* < 0.01, ***, *p* < 0.001 compared to the sham group or unilateral TTT group.

HE staining results showed a growing granulation tissue filling the wound cavity and epidermal regeneration in the unilateral and bilateral groups versus the sham group, and these changes in samples at 14 days were more visible than these at 7 days (Figure 2A). Additionally, Masson’s trichrome staining revealed a significant increase in collagen fibers in wound skins, suggesting that TTT surgery significantly promotes collagen fiber content in wound skins (Figure 2B). Immunofluorescence results for CD31 and VEGFA expression also showed that the unilateral group was stronger than the sham, and the bilateral stronger than the unilateral group. TTT significantly increased CD31 and VEGFA expression, with fluorescence intensity at 14 days stronger than that at 7 days, indicating enhanced angiogenesis (Figure 2C and D). Overall, these results suggest that TTT can increase angiogenesis and collagen fiber formation, promoting wound epithelialization.

### The impact of TTT on EPC mobilization and homing

**Figure 3.**
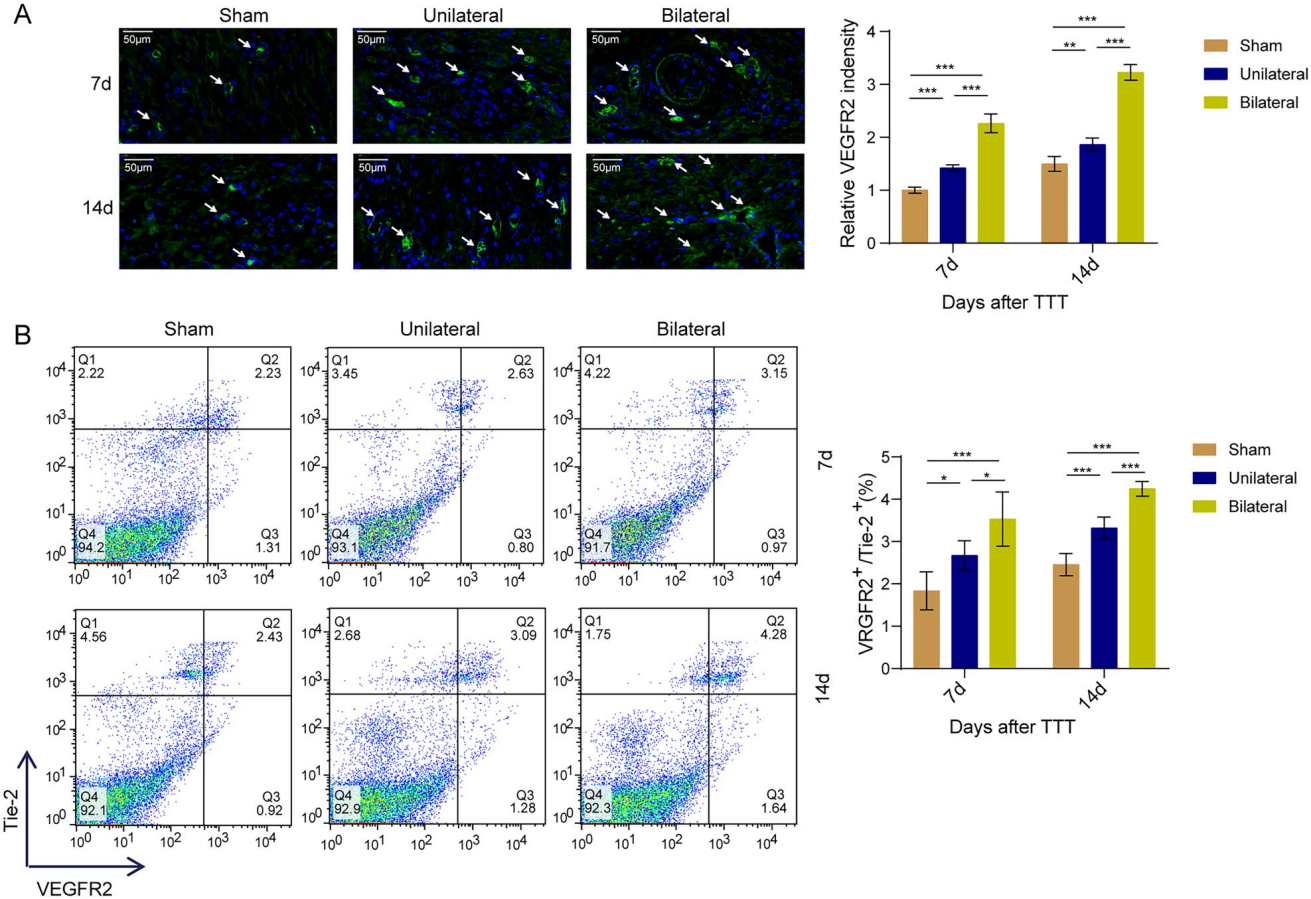
The impact of TTT on EPC mobilization and homing. (A) Immunofluorescence for VEGFR2 expression, anti-VEGFR2 antibody and DAPI to the nuclei, and the arrow to indicate positive staining. Scale bar = 50 μm. (B) Flow cytometry analysis of peripheral blood VEGFR2/Tie-2 positive ratio. *, *p* < 0.05, **, *p* < 0.01, ***, *p* < 0.001 compared to the sham group or unilateral TTT group.

Based on previous studies and our preliminary conclusions that TTT promotes angiogenesis and wound healing, we further explored the impact of TTT on EPC mobilization and homing. Immunofluorescence results showed that one of the key factors involved in EPCs, VEGFR2, was significantly increased at 7 and 14 days post-TTT, with 14-day expression higher than at 7 days (Figure 3A). Flow cytometry also revealed that compared to the sham and unilateral TTT groups, the bilateral TTT group showed a significant increase in VEGFR2/Tie-2 positive ratio at days 7 and 14 post-surgery (Figure 3B). These results suggest that TTT may promote angiogenesis and wound healing by inducing EPC mobilization and homing.

### The effect of TTT on VEGFA and CXCL12 expression

**Figure 4.**
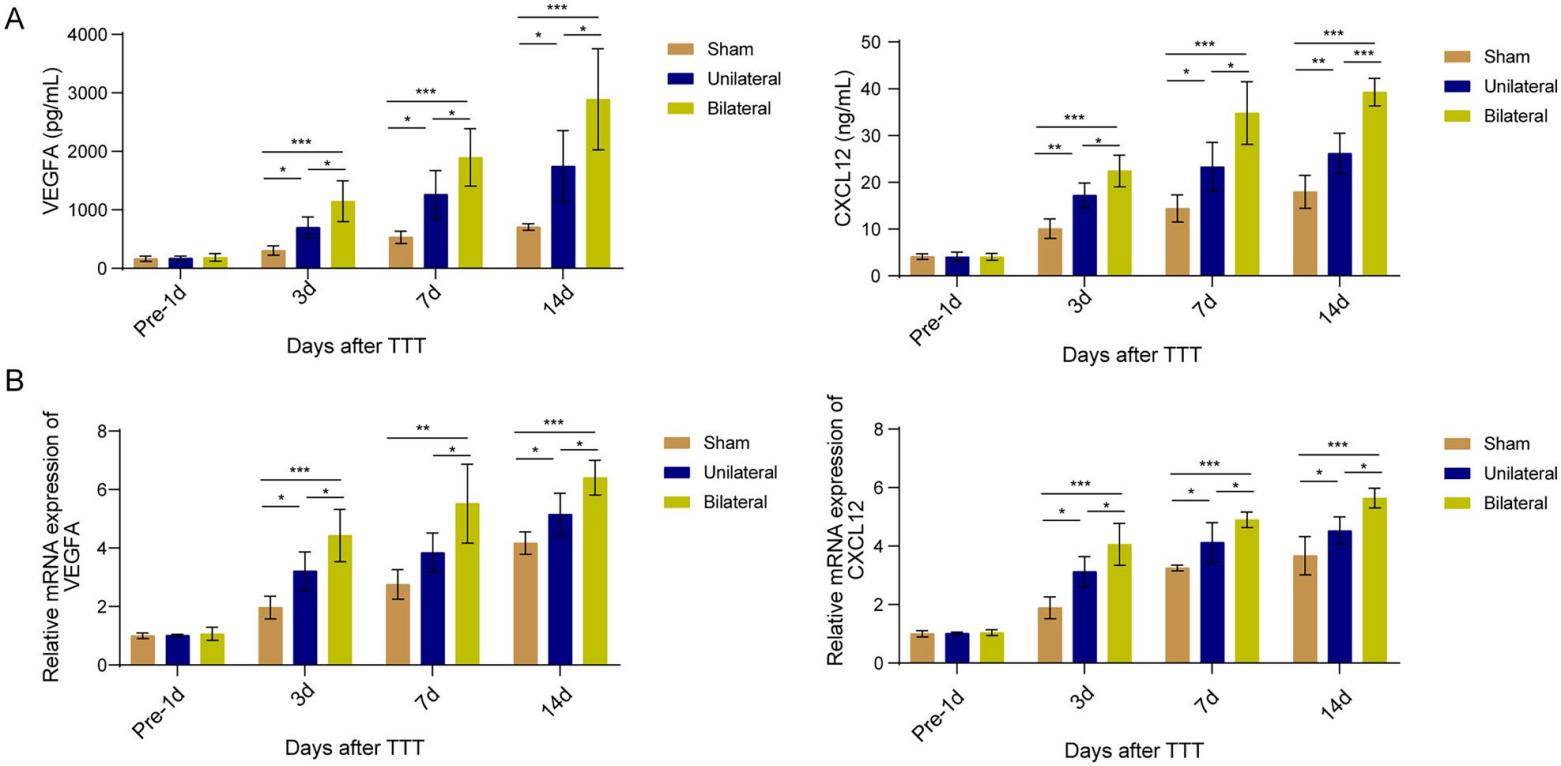
The effect of TTT on VEGFA and CXCL12 expression (A) ELISA for serum levels of VEGFA and CXCL12. (B) qPCR for skin tissue expression of VEGFA and CXCL12. *, *p* < 0.05, **, *p* < 0.01, ***, *p* < 0.001 compared to the sham group or unilateral TTT group.

Previous research has identified VEGFA and CXCL12 as key molecules in the mobilization and homing of EPCs, angiogenesis, and wound healing processes [24]. VEGFA promotes the mobilization and migration of EPCs to damaged tissues by activating specific receptors, participating in the formation of new blood vessels [25]. CXCL12, as a chemokine, attracts EPCs to ischemic or damaged areas, promoting vascular repair and tissue regeneration. The synergistic action of these two molecules is crucial for wound healing and angiogenesis. Therefore, we hypothesized that TTT might promote angiogenesis and wound healing by affecting the expression of VEGFA and CXCL12. Subsequent analysis of serum levels of VEGFA and CXCL12 revealed that TTT significantly increased the levels of these molecules (Figure 4A). Moreover, analysis of RNA expression in skin tissues confirmed that TTT significantly upregulated the mRNA expression levels of VEGFA and CXCL12 (Figure 4B). Thus, we can confirm that TTT may promote angiogenesis and wound healing by inducing wound fibroblasts to release VEGFA and CXCL12, mediating EPC mobilization and homing.

## Discussion

Our study focuses on DFUs, a serious complication in diabetic patients that can lead to lower limb amputations. Current treatments for DFUs, such as off-loading, surgical debridement, and skin grafting, often have limited effectiveness, especially for ulcers classified under higher Wagner grades [1]. This study introduces TTT as a novel approach for treating DFUs. It hypothesizes that TTT stimulates the mobilization and homing of EPCs, thereby promoting wound healing through angiogenesis [18]. TTT, as a method for treating DFUs, has demonstrated its effectiveness in numerous studies. These studies indicate that TTT can accelerate the healing of ulcers, reduce the risk of infection, and improve the quality of life for patients [26,27]. Our findings also showed that both unilateral and bilateral TTT surgeries significantly enhance wound healing in diabetic rabbit foot ulcers. Notably, TTT surgery can considerably shorten the wound healing time, demonstrating a significant positive impact on the healing process of DFUs.

A previous research has shown the critical role of angiogenesis in the wound healing process, which underscores the importance of effective wound management under diabetic conditions [6]. And, research has found that TTT can accelerate the healing of diabetic foot ulcers by promoting angiogenesis and regulating immune responses [17]. Additionally, studies have discovered the role of mesenchymal stem cells (MSCs) in TTT surgery, particularly in promoting neovascularization and wound healing [28]. What’s more, in our study, we found that TTT promotes vascular growth and recovery, significantly increasing the collagen fiber content in wound skins.

EPCs are a type of stem cell with the potential for angiogenesis, playing a crucial role in vascular formation and repair during wound healing [29]. And, Zhao et al.’s study emphasizes the role of EPCs in wound healing, particularly in angiogenesis and collagen fiber deposition [30]. Research has found that EPCs play a significant role in the treatment and management of diabetic foot, contributing to angiogenesis, wound healing, and the treatment of DFUs. Moreover, as a crucial source of endothelial cells, EPCs may play a key role in the pathophysiology of diabetic foot, especially in processes related to vascular health and repair [31]. Consistently, our study found that TTT significantly increases the ratio of VEGFR2/Tie-2 positive cells in peripheral blood and VEGFR2 expression in wound skins, indicating that TTT promotes EPC mobilization and homing.

A Research has found that factors, such as VEGFA and CXCL12, can influence the function of EPCs and the process of angiogenesis, revealing that the therapeutic potential of EPCs in promoting angiogenesis can be enhanced by regulating these chemokines [32]. VEGFA and CXCL12 are key molecules for EPC mobilization and homing, crucial for angiogenesis and the wound healing process. Additionally, studies have revealed the impact of the CXCL13/CXCR5 axis on the homing and angiogenesis of EPCs in the progression of rheumatoid arthritis (RA), particularly highlighting the role of CXCL12 in EPCs, especially in promoting angiogenesis and the migration of EPCs in inflammatory environments [33]. Furthermore, GDF11 promotes wound healing in diabetic mice by stimulating HIF-1α-VEGF/SDF-1α-mediated EPC mobilization and neovascularization [13].

Unlike the study by Qin et al. [34] which explored the application of TTT technology in the treatment of DFUs using a rat model, we used New Zealand white rabbits to focus on promoting angiogenesis and collagen fiber formation. The rat study placed greater emphasis on immune modulation, particularly the transition of macrophages from M1 to M2. Our research provides a new perspective through the rabbit models, further validating the effectiveness of TTT technology in different animal models, and highlighting its significant role in angiogenesis and tissue repair.

The results of our study show that TTT significantly elevates the expression levels of VEGFA and CXCL12, suggesting that TTT promotes wound healing by enhancing the expression of these molecules, thereby facilitating EPC mobilization and homing. Although TTT shows potential in the treatment of DFUs, further research is needed on its best clinical practices, mechanisms of action, and synergistic effects with other treatments. For instance, the timing and intensity of EPC mobilization by TTT, the mechanisms of EPCs in DFUs, and how TTT can be combined with traditional treatments to improve efficacy are all important areas for future research [35].

## Conclusions

**Figure 5.**
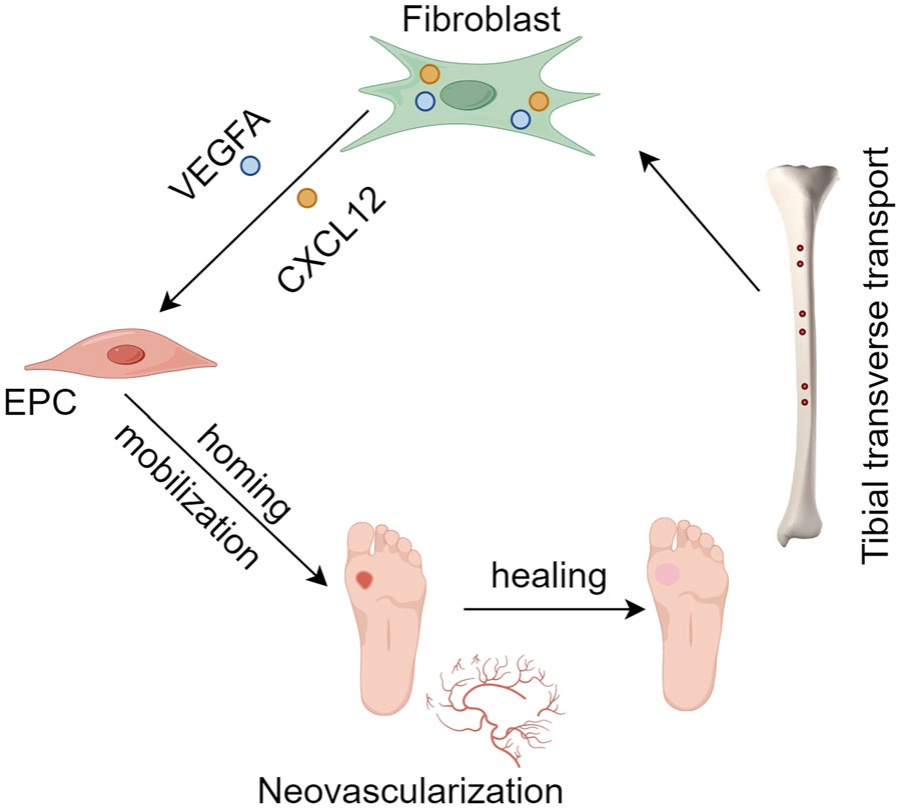
Mechanism diagram of TTT promoting wound healing in DFUs (by figdraw). TTT induces wound fibroblasts to release VEGFA and CXCL12, thereby mediating EPC mobilization and homing, promoting angiogenesis and wound healing.

The study demonstrates that TTT significantly enhances the healing of diabetic rabbit foot ulcers, primarily by promoting angiogenesis, collagen fiber formation, mobilization and homing of EPCs, and upregulating the expression of VEGFA and CXCL12 (Figure 5). These findings highlight the potential therapeutic efficacy and mechanisms of TTT in treating DFUs.

## Data Availability

Not applicable

## Data availability statement

The datasets used or analyzed during the current study are available from the corresponding author on reasonable request.
